# Separate Origins of Group I Introns in Two Mitochondrial Genes of the Katablepharid *Leucocryptos marina*

**DOI:** 10.1371/journal.pone.0037307

**Published:** 2012-05-11

**Authors:** Yuki Nishimura, Ryoma Kamikawa, Tetsuo Hashimoto, Yuji Inagaki

**Affiliations:** 1 Graduate School of Life and Environmental Sciences, University of Tsukuba, Tsukuba, Japan; 2 Center for Computational Sciences, University of Tsukuba, Tsukuba, Japan; University of Melbourne, Australia

## Abstract

Mitochondria are descendants of the endosymbiotic α-proteobacterium most likely engulfed by the ancestral eukaryotic cells, and the proto-mitochondrial genome should have been severely streamlined in terms of both genome size and gene repertoire. In addition, mitochondrial (mt) sequence data indicated that frequent intron gain/loss events contributed to shaping the modern mt genome organizations, resulting in the homologous introns being shared between two distantly related mt genomes. Unfortunately, the bulk of mt sequence data currently available are of phylogenetically restricted lineages, *i.e.*, metazoans, fungi, and land plants, and are insufficient to elucidate the entire picture of intron evolution in mt genomes. In this work, we sequenced a 12 kbp-fragment of the mt genome of the katablepharid *Leucocryptos marina*. Among nine protein-coding genes included in the mt genome fragment, the genes encoding cytochrome *b* and cytochrome *c* oxidase subunit I (*cob* and *cox1*) were interrupted by group I introns. We further identified that the *cob* and *cox1* introns host open reading frames for homing endonucleases (HEs) belonging to distantly related superfamilies. Phylogenetic analyses recovered an affinity between the HE in the *Leucocryptos cob* intron and two green algal HEs, and that between the HE in the *Leucocryptos cox1* intron and a fungal HE, suggesting that the *Leucocryptos cob* and *cox1* introns possess distinct evolutionary origins. Although the current intron (and intronic HE) data are insufficient to infer how the homologous introns were distributed to distantly related mt genomes, the results presented here successfully expanded the evolutionary dynamism of group I introns in mt genomes.

## Introduction

Group I (gI) introns are a major class of introns found in bacterial genomes, mitochondrial and plastid genomes, and eukaryotic nuclear genomes [1] as well as genomes of viruses/phages [2]. In eukaryotes, gI introns in the nuclear genomes are exclusively inserted in ribosomal RNA (rRNA) genes, whereas the introns reside in genes encoding both structural RNAs and proteins in organellar genomes [3]. The typical secondary structure of gI introns consists of approximately 10 double helical elements designated as P1–P10 [4]. These helical elements are further organized into three domains at the tertiary structural level, which are important for efficient splicing of this class of introns [5]. Many gI introns host open reading frames (ORFs) for homing endonucleases (HEs) [6], which may facilitate intron invasion into the intron-less alleles within a population of the same species, as well as those in different species [7], [8], [9]. Intron-encoded (intronic) HEs are divided into four types, such as LAGLIDADG, GIY-YIG, His-Cys box, and NHN families, on the basis of highly conserved motifs [10].

Mitochondrial (mt) gene/genome data are potentially informative for inspecting the evolution of gI introns hosting HEs, as a number of gI introns has been identified in mt genomes [4]. Nevertheless, the mt intron data currently available are largely derived from restricted taxonomic groups, such as metazoans, fungi, and members of Viridiplantae (land plants plus green algae), and our knowledge regarding the evolution of gI introns is highly likely incomplete due to general research interests biased toward ‘popular’ taxa in biological sciences. For instance, the mt genomes of the three groups mentioned above have been more intensively sequenced than other eukaryotic groups (except metazoan)—2,541 out of 2,782 completed mt genomes are of metazoans, and 97 and 61 mt genomes are of fungi and land plants/green algae amongst 241 non-metazoan mt genomes listed in NCBI Entrez Genome database (http://www.ncbi.nlm.nih.gov/sites/entrez?db=genome), as of January 2012. Thus, we can shed light on novel aspects in the evolution of gI introns in mt genomes by investigating the intron data from the lineages of which mt gene sequences have not been accumulated.

Katablepharida is a group of heterotrophic unicellular eukaryotes whose members widely distribute in aquatic environments [11]. Phylogenetic analyses of small and large subunit rRNA genes strongly suggested that katablepharids are closely related to cryptomonads and goniomonads [12]. In this study, we report two gI introns hosting LAGLIDADG-type HEs in the katablepharid *Leucocryptos marina* mt genome, and explored the evolutionary histories of these introns by combining their putative secondary structures, the intron positions, and the phylogenetic affinities of the intronic HEs.

## Results and Discussion

### Group I introns in *Leucocryptos* mt genome

**Figure 1:**
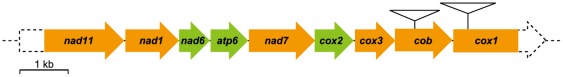
Primary structure of the partial mitochondrial genome of the katablepharid *Leucocryptos marina*. Protein-coding genes (and their directions) are shown by arrows. Abbreviations; *nad11*, NADH dehydrogenase subunit 11; *nad1*, NADH dehydrogenase subunit 1; *nad6*, NADH dehydrogenase subunit 6; *atp6*, ATP synthase F0 subunit 6; *nad7*, NADH dehydrogenase subunit 7; *cox2*, cytochrome *c* oxidase subunit 2; *cox3*, cytochrome *c* oxidase subunit 3; *cob*, cytochrome *b*; *cox1*, cytochrome *c* oxidase subunit 1. Introns inserted in the *cob* and *cox1* genes are shown as triangles. The genes initially amplified by reverse transcriptase PCR are shown in orange, while those amplified from genomic DNA were in green. The 5′ terminus of the *nad11* gene and the 3′ terminus of the *cox1* gene were not determined in the current study (highlighted by dotted lines).

We determined an approximately 12 kbp-long region of the *Leucocryptos* mt genome including NADH dehydrogenase subunit 11 (*nad11*), NADH dehydrogenase subunit 1 (*nad1*), NADH dehydrogenase subunit 6 (*nad6*), ATP synthase F0 subunit 6 (*atp6*), NADH dehydrogenase subunit 7 (*nad7*), cytochrome *c* oxidase subunit 2 (*cox2*), cytochrome *c* oxidase subunit 3 (*cox3*), cytochrome *b* (*cob*), and cytochrome *c* oxidase subunit 1 (*cox1*) genes in this order (Fig. 1; Note that the 3′ terminus of *cox1* and the 5′ terminus of *nad11* are not available in this mt genome fragment). The intergenic spacer regions are short, ranging from 4–65 bp in length. Neither transfer RNA nor ribosomal RNA gene was found in the mt genome fragment determined in the current study. By the comparison between the cDNA and genomic sequences, two introns in this region, one in the *cob* gene and the other in the *cox1* gene, were detected (highlighted as triangles in Fig. 1). No sign of RNA editing was found so far.

**Figure 2:**
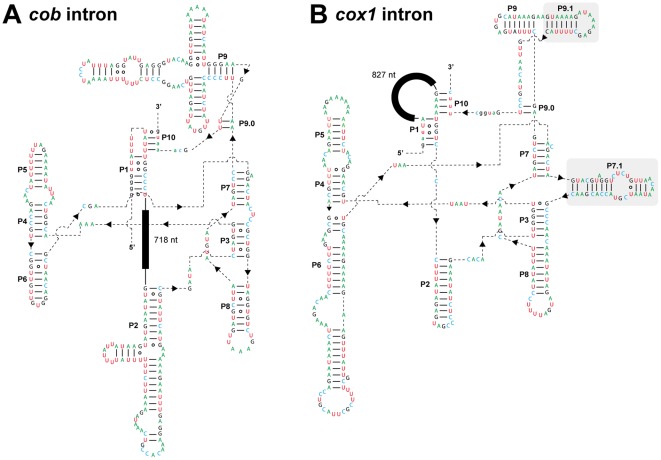
Putative secondary structures of the group I intron RNAs. **A.** Secondary structure of the *Leucocryptos cob* intron. Putative Watson–Crick and wobble base pairs are shown by lines and open circles, respectively. Capital and small letters represent intron and exon nucleotides, respectively. Double helical structures, which are characteristic to group I introns, are labeled as P1–P10. The open reading frame (ORF) for a LAGLIDADG-type homing endonuclease (closed box; 217 amino acid residues) was found in the 718 nucleotide-long loop region between P1 and P2. **B.** Secondary structure of the *Leucocryptos cox1* intron. The details of this figure are same as described in A, except the ORF for a LAGLIDADG-type homing endonuclease (closed box; 267 amino acid residues) was found in the 827 nucleotide-long loop region between P1 and P10. P9.1 and P7.1, which are absent in the *Leucocryptos cob* intron, are shaded.

The two introns in the *cob* and *cox1* genes are likely of group I, as these sequences can be folded into typical secondary structures of gI introns comprising 11–12 double helical domains (P1–P10; Figs. 2A & 2B). In our BlastN survey, the putative core region of the *cob* intron showed sequence similarity to those of group ID introns [e.g., the one lying in the *Chaetosphaeridium globsum cob* gene (GenBank accession no. AF494279.1) with an *E*-value = 10^−13^]. On the other hand, the putative core region of the *cox1* intron appeared to share sequence similarity to those of group IA1 introns [e.g., the one lying in the *Scenedesmus obliqus* large subunit of rRNA gene (GenBank accession number AF204057.1) with an *E*-value = 2×10^−6^]. The two gI introns are also distinguishable from one another by the two following points: (i) The *cox1* intron have two extra double helical domains, P7.1 and P9.1 (shaded in Fig. 2A), which are absent in the *cob* intron; (ii) An ORF occupies the loop region between P1 and P10 in the *cob* intron, while an ORF places in the loop region between P1 and P2 in the *cox1* intron (Figs. 2A & 2B)

The ORFs hosted in the *cob* intron and *cox1* intron likely encode 217 amino acid (aa) residue-long and 267 aa residue-long polypeptides, respectively. The two intronic ORFs likely encode LAGLIDADG-type HEs, but no significant similarity was detected between their putative aa sequences by a BlastP search (bl2seq) with the default settings. Henceforth here, we designate the HE hosted in the *cob* intron as HE*^Lm-cob^*, while that hosted in the *cox1* intron as HE*^Lm-cox1^*. HE*^Lm-cob^* and HE*^Lm-cox1^* appeared to belong to distant superfamilies, LAGLIDADG_2 (pfam031611) and LAGLIDADG_1 (pfam00961), respectively.

### Origin of the *Leucocryptos cob* intron

**Figure 3:**
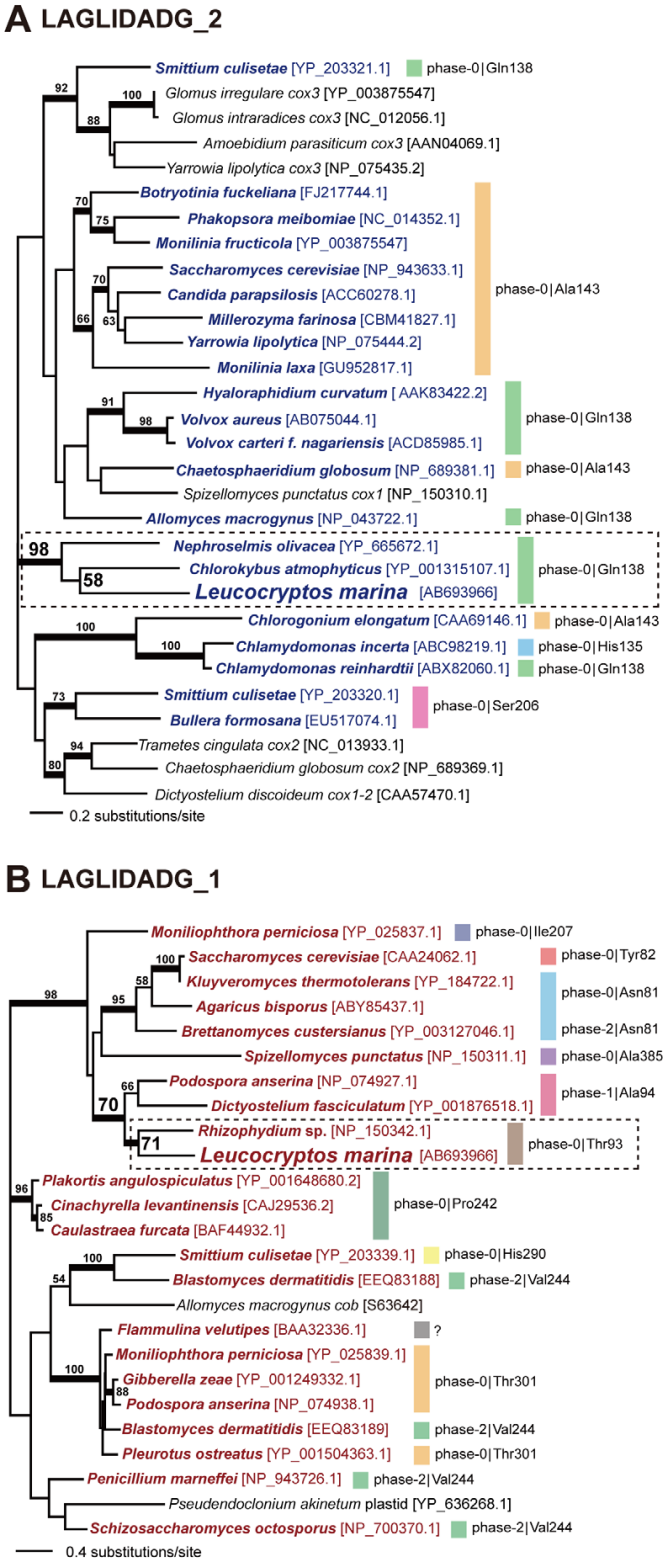
Maximum-likelihood (ML) phylogenetic analyses of homing endonuclease (HE) sequences. **A.** Unrooted ML phylogeny inferred from the LAGLIDADG_2 alignment containing 183 amino acid positions. Thirty HEs belonging to LAGLIDADGE_2 superfamily were subjected to the ML and Bayesian methods. The HEs hosted in *cob* introns are shown in dark blue. The details of the homing positions of the HE-hosting *cob* introns (phase and codon) are given on the right side of the tree. Codon numbers are based on the *Saccharomyces cerevisiae cob* gene (GenBank accession number NC_001224). Only ML bootstrap values equal to or greater than 50% are shown. The resultant tree inferred from Bayesian analysis was essentially identical to that from the ML analysis (data not shown). The branches supported by Bayesian posterior probabilities (BPPs) equal to or greater than 0.95 were highlighted by thick lines. The GenBank accession numbers of the HE sequences used in this tree are given in brackets. **B.** Unrooted ML phylogeny inferred from the LAGLIDADG_1 alignment containing 191 amino acid positions. Twenty five HEs belonging to LAGLIDADGE_1 superfamily were subjected to the ML and Bayesian methods. The HEs hosted in *cox1* introns are shown in dark red. The details of the homing positions of the HE-hosting *cox1* introns (phase and codon) are given on the right side of the tree. Codon numbers are based on the *S. cerevisiae cox1* gene (GenBank accession number NC_001224). We are unsure the precise position of the intron identified in the *Flammulina velutipues cox1* gene, as only HE sequence has been deposited in the GenBank database (labeled with a question mark). Other details are same as described in A.

We aligned the aa sequences of 30 members of LAGLIDADG_2 superfamily including HE*^Lm-cob^*, and subjected to the maximum-likelihood (ML) and Bayesian phylogenetic analyses. In the unrooted phylogeny of this ‘LAGLIDADG_2’ alignment, HE*^Lm-cob^* and two HEs encoded in the *cob* introns of two green algae, *Nephroselmis olivacea* and *Chlorokybus atmophyticus*, grouped together with a BP of 98% and a Bayesian posterior probability (BPP) of 1.00 (surrounded by dotted line in Fig. 3A), suggesting that the *cob* introns harboring the *Leucocryptos* and green algal HEs evolved from a single ancestral intron. The ancestral intron most likely (i) lied at phase-0 position of the codon corresponding to Gln138 in the *Saccharomyces cerevisiae cob* gene (GenBank accession number NC_001224), and (ii) hosted a HE belonging to LAGLIDADG_2 superfamily in the loop region between P1 and P2 as shown in Fig. S1A. Unfortunately, it is difficult to retrieve deeper insights for the origin of *Leucocryptos cob* intron by intron positions, as the HEs hosted by the introns lying in the homologous positions were sporadically distributed in the LAGLIDADG_2 phylogeny (Fig. 3A).

### Origin of the *Leucocryptos cox1* intron

We prepared a ‘LAGLIDADG_1’ alignment comprising the aa sequences of HE*^Lm-cox1^* and 24 members of LAGLIDADG_1 superfamily. The unrooted LAGLIDADG_1 phylogeny united HE*^Lm-cox1^* and the HE hosted in the forth out of 15 *cox1* introns in the fungus *Rhizophydium* sp. with a BP of 71% and a BPP of 0.96 (surrounded by dotted line in Fig. 3B). Although the statistical support for this clade was inconclusive, the introns hosting the two HEs described above exclusively share the homing position—phase-0 position of the codon corresponding to Thr93 in the *S. cerevisiae cox1* gene (GenBank accession number NC_001224). Thus, *Leucocryptos cox1* intron and the forth intron in *Rhizophydium cox1* gene likely derived from a single ancestral intron, which lied at phase-0 position of the codon corresponding to Thr93 in the *S. cerevisiae cox1* gene, and hosted a LAGLIDADG_1-type HE in the loop region between P1 and P10 (see Fig. S1B). Of note, independent from the *Leucocryptos cox1* intron, *cox1* introns in multiple land plant species appeared to share the ancestries with the fungal introns [13], [14]. Thus, fungal mt genomes might hold keys to elucidate the evolution in mt introns as a whole.

The clan of HE*^Lm-cox1^* and the *Rhizophydium* HE was further connected to the HE encoded in the first out of 16 *cox1* intron of the fungal *Podospora anserina*, and that encoded in a single *cox1* intron of the mycetozoan *Dictyostelium fasciculatam* (BP of 70% and BPP of 0.99; Fig. 3B). Both *Podospora* and *Dictyostelium* introns lie between the first and second letters of the codon corresponding Ala94 in the *S. cerevisiae cox1* gene (phase-1), being in close proximity to but apparently distinct from the homing position of the *Leucocryptos* and *Rhizophydium* introns (see above). One possibility is that HE*^Lm-cox1^* and the *Rhizophydium* HE, and the *Podospora* and *Dictyostelium* HEs have evolved from a single ancestral HE and still recognize the identical nucleotide sequence (or very similar sequences), but the cleavage position altered after the separation of the two HE pairs. In any case, the evolutionary link between the *cox1* introns in *Leucocryptos* and *Rhizophydium*, and those in *Podospora* and *Dictyostelium* can be assessed only after the enzymatic properties of the HEs hosted in the four *cox1* introns are characterized.

### Intron evolution in *Leucocryptos* mt genome: ‘lateral transfer’ versus ‘parallel loss’

Introns in organellar genomes are generally regarded as mobile genetic elements powered by intronic HEs, as ‘trans-genomic’ intron invasion have been accumulated in the literature [9]. In the global eukaryotic phylogeny, katablepharids highly likely form a clade with goniomonads and cryptomonads, but are closely related to neither green algae nor fungi [12], [15]. Thus, the evolutionarily homologous introns resided in distantly related mt genomes (Figs. 2A & 2B) can be rationalized by lateral transfer events. Nevertheless, considering the cyclic model for gain and loss of selfish genetic elements including gI introns [16], we cannot exclude the alternative scenario which assumes that (i) the two introns in *cob* and *cox1* genes discussed above have been vertically inherited from the common ancestor of katablepharids, green algae and fungi, but (ii) secondary intron loss occurred in other descendent lineages, as the HE sequences considered here unlikely represent the true diversity of LAGLIDADG_2-type or LAGLIDADG_1-type HE superfamily. The origins of the two gI introns found in *Leucocryptos* mt genome should be revisited after in-depth surveying introns and intronic HEs in the mt genomes of phylogenetically broad eukaryotic lineages, particularly those of close relatives of katablepharids, such as goniomonads and cryptomonads.

## Materials and Methods

### From cell culture to DNA sequencing

The katablepharid *Leucocryptos marina* NIES-1335 and the haptophyte *Chrysochromulina* sp. NIES-1333 were purchased from the National Institute for Environmental Study (NIES). *Leucocryptos* was maintained in f/2 medium (http://mcc.nies.go.jp/02medium.html#f2) with *Chrysochromulina* as a prey at 20°C under 14∶10 hours of light∶dark cycles.

We harvested the *Leucocryptos* cells containing a small amount of the *Chrysochromulina* (prey) cells, and this sample was then subjected to DNA and RNA extractions by using Plant DNA Isolation Reagent (TaKaRa) and RNeasy Plant Mini Kit (QIAGEN), respectively. Total RNA was used for random hexamer-primed cDNA synthesis by Superscript II reverse transcriptase (Invitrogen). The experiments mentioned above were conducted by following the manufactures' instructions. The cDNA and total DNA were used as the templates for polymerase chain reactions (PCR) aiming at the amplification of gene transcripts and genome fragments, respectively.

We initially amplified six mt gene transcripts by reverse transcriptase PCR (RT-PCR) with the primer sets shown in Table 1—*cob*, *cox1*, *cox3*, *nad1*, *nad7*, and *nad11*. PCR products were cloned into pGEM-TEasy vector (Promega). For each gene transcript, we completely sequenced eight clones and confirmed no sequence heterogeneity among clones, except the *cob* and *cox3* transcripts. The *cob* and *cox3* samples appeared to consist of two distinctive types of amplicons, one with and the other without in-frame TGA codons (data not shown; no in-frame TGA codon was found in the *cox1*, *nad1*, *nad7*, or *nad11* sample). We regarded the amplicons with in-frame TGA codons as the mt gene transcripts from the haptophyte (*Chrysochromulina*) prey cells for two reasons: Firstly, our preliminary phylogenetic analyses indicated that the two amplicons were distantly related to each other, and only the one with in-frame TGA codons displayed an intimate affinity to the haptophyte homologues (Figs. S2A & B). Secondly, the genus *Chrysochromulina* belongs to one of the two classes in Haptophyta, Prymnesiophyceae, whose mt genomes assign TGA, one of the three termination codons in the standard genetic code, to tryptophan [17], [18]. On the basis of the phylogenetic results and feature in codon usage, we concluded that the *cob* and *cox3* transcripts with in-frame TGA codons were most likely originated from the haptophyte prey cells, and were not considered in the following experiments.

We then amplified the mt genome fragments corresponding to the six transcripts with no in-frame TGA codon with exact-match primers (not shown). We also amplified the intergenic spacer regions between *nad11* and *nad1*, *nad1* and *nad7*, *nad7* and *cox3*, *cox3* and *cob*, and *cob* and *cox1* with outwarded exact match primers designed based on the six mt gene transcripts initially determined (see above) as performed in previous works [19], [20]. As the result of the PCR with outward primers, three genes (*nad6*, *atp6*, and *cox2*) were additionally found. Cloning and sequencing were performed as described above. The partial mt genome sequence was deposited to DNA Data Bank of Japan (GenBank/EMBL/DDBJ accession no. AB693966).

### Prediction of intron secondary structures

Each of *cob* and *cox1* genes in the *Leucocryptos* mt genome appeared to possess a single gI intron with a HE (see Results and Discussion). Both 5′ and 3′ splice sites were determined by comparing the cDNA and genomic sequences. Intron secondary structures were predicted using MFOLD [21], followed by manual modification by referring the general structures of gI introns presented in GOBASE [22].

**Table 1:** Degenerate primers used for reverse-transcription PCR.

Genes	Names	Directions	Sequences (5′ – 3′)
*Cob*	HcobF	forward	GNGAYGTNAAYAAYGGNTGG
	HcobR	reverse	ACDATRTGNGCNGGNGTNACC
*cox1*	Hcox1F	forward	ACNAAYCAYAARGAYATHGG
	Hcox1R	reverse	NACNCCNACRAANGTRCACC
*cox3*	Hcox3F	forward	CCNTTYCAYTTRGTNGAYCC
	Hcox3R	reverse	NACNACRTCNACRAARTGCC
*nad1*	Hnad1F	forward	CGNGGNCCNAAYGTNGTNGG
	Hnad1R	reverse	NARYTCNGCYTCNGCYTCNGG
*nad7*	Hnad7F	forward	AAYTTYGGNCCNCARCAYCC
	Hnad7R	reverse	NACNCCRAAYTCNCCYTTNG
*nad11*	Hnad11F	forward	GTNGCNGGNAAYTGYKGNATG
	Hnad11R	reverse	NGTNARNGCNCCNACNGGRCA

### Phylogenetic analyses of intronic HEs

The HE encoded in the *Leucocryptos cob* intron (HE*^Lm-cob^*) was aligned with 29 HEs belonging to the LAGLIDADG_2 superfamily, which showed significant similarity to HE*^Lm-cob^* in TBlastN search against the GenBank nr database (*E*-values<10^−10^). We carefully assessed the alignments from the Blast search, and excluded redundant sequences and the sequences which produced very short alignments with HE*^Lm-cob^*. After manual refinement followed by the exclusion of ambiguously aligned positions, 183 aa positions were remained in the final ‘LAGLIDADG_2’ alignment. Pairwise aa identities and similarities ranged from 30 to 98%, and from 48 to 98%, respectively (Fig. S3A). The HE sequence hosted in the *Chlorokybus atmophyticus cob* intron showed the highest aa identity (47%) to HE*^Lm-cob^*, while the ones hosted in the *Millerozyma farinose* and *Chlorogonium elongatum cob* genes showed the lowest aa identity (34%) to HE*^Lm-cob^* (see the upper triangular in Fig. S3A). The HE sequence hosted in the *Chlorokybus atmophyticus cob* intron showed the highest aa similarity (65%) to the HE*^Lm-cob^*, while the one hosted in the *Chlamydomonas incerta cob* genes showed the lowest aa similarity (49%) to HE*^Lm-cob^* (see the lower triangular in Fig. S3A).

The same procedure described above was repeated to prepare a ‘LAGLIDADG_1’ alignment including the HE encoded in the *Leucocryptos cox1* intron (HE*^Lm-cox1^*) and 24 HEs belonging to the LAGLIDADG_1 superfamily, which can form global alignments with HE*^Lm-cox1^* with TBlastN *E*-values<10^−10^. The final LAGLIDADG_1 alignment contains 191 unambiguously-aligned aa positions. Pairwise aa identities and similarities ranged from 14 to 95%, and from 34 to 97%, respectively (Fig. S3B). The HE sequence hosted in the *Rhizophydium* sp. *cox1* intron showed the highest aa identity (44%) to HE*^Lm-cox1^*, while the one hosted in the *Flammulina velutipes cox1* gene showed the lowest aa identity (20%) to HE*^Lm-cox1^* (see the upper triangular in Fig. S3B). The HE sequence hosted in the *Podospora anserina cox1* intron showed the highest aa similarity (62%) to HE*^Lm-cox1^*, while the ones hosted in the *Blastomyces dermatitidis cox1* gene and *Allomyces macrogynus cob* gene showed the lowest aa similarity (41%) to HE*^Lm-cob^* (see the lower triangular in Fig. S3B). The GanBank accession numbers of the HE sequences considered in the two alignments, and the precise positions of the introns hosting these HEs are shown in Figs. 3A and B.

The two HE alignments were separately subjected to ML analysis. The LG model [23] incorporating empirical aa frequencies and among-site rate variation approximated by a discrete gamma (Γ) distribution with four categories (LG+Γ+F model) was selected as the most appropriate model for the aa substitutions in the LAGLIDADG_1 alignment by the program Aminosan [24] under the Akaike information criterion. Similarly, the VT model [25] incorporating empirical aa frequencies and among-site rate variation approximated by a discrete Γ distribution with four categories (VT+Γ+F model) was selected as the most appropriate model for the aa substitutions in the LAGLIDADG_2 alignment. The ML analyses were performed using RAxML 7.2.1 [26] with the selected model described above. The ML tree was heuristically searched from 10 distinct parsimony trees. In RAxML bootstrap analyses (100 replicates), the heuristic tree search was performed from a single parsimony tree per replicate.

The two HE alignments were also analyzed by Bayesian method with the LG+Γ model using PhyloBayes v.3.2 [27]. As VT model is not available in PhyloBayes, we applied the LG+Γ model to the LAGLIDADG_2 alignment. Two independent Markov chain Monte Carlo chains (MCMC) were run for 72,000–78,000 points. The first 100 points were discarded as ‘burn-in’ on the basis of the log-likelihood plots (data not shown). For each analysis, we compared the frequencies of all bipartitions observed in the two independent MCMC runs in detail, and confirmed the convergence between the two runs by the ‘maxdiff’ value being smaller than that recommended in the manual of the program (i.e., maxdiff<0.1; data not shown). Subsequently, the consensus trees with branch lengths and BPP were calculated from the rest of the sampling trees.

## Supporting Information

Figure S1 **Putative secondary structures of group I intron RNAs.** **A.** Schematic structures of *Leucocryptos*, *Chlorokybus*, and *Nephroselmis cob* introns. LAGLIDADG_2-type homing endonucleases are encoded in the region between P1 and P2 in the three introns (shown as closed boxes). **B.** Schematic structures of *Leucocryptos* and *Rhizophydium cox1* introns. Both introns harbor LAGLIDADG_1-type homing endonucleases in the region between P1 and P10 (shown as closed boxes).
(TIF)

Figure S2 **Maximum-likelihood (ML) analyses of the COB and COX3 amino acid (aa) alignments.** **A.** The ML phylogeny inferred from the COB alignment comprises 31 taxa with 368 unambiguously aligned aa positions **B.** The ML phylogeny inferred from the COX3 alignment comprising 26 taxa with 218 unambiguously aligned aa positions. *Leucocryptos marina* and *Chrysochromulina* sp. are highlighted by arrowheads. The haptophyte clade is shaded. Only ML bootstrap values equal to or greater than 50% are shown. **Methods:** The two aa alignments were separately analyzed with the ML method with the LG+Γ+F model by using RAxML ver. 7.2.1. The details of the ML and ML bootstrap analyses were same as described in Materials and Methods/Phylogenetic analyses of intronic HEs.
(TIF)

Figure S3 **Amino acid (aa) sequence homology.** **A.** Pairwise aa identity matrix of the 30 endonuclease (HE) sequences in the LAGLIDADG_2 alignment. We also recoded 20 aa characters in the HE sequences to six Dayhoff classes, and then made the identity matrix presented below diagonal. **B.** Pairwise aa identity matrix of the 25 HE sequences in the LAGLIDADG_1 alignment. We also provide the pairwise ‘Dayhoff-class’ identity matrix below diagonal. For each sequence, the GenBank accession no. is shown in brackets.
(TIF)
