# Supplementary figures and images for: Separate Origins of Group I Introns in Two Mitochondrial Genes of the Katablepharid Leucocryptos marina
Source: PLoS One. 2012 May 11;7(5):e37307. doi: 10.1371/journal.pone.0037307 (PMC3350498; doi:10.1371/journal.pone.0037307)

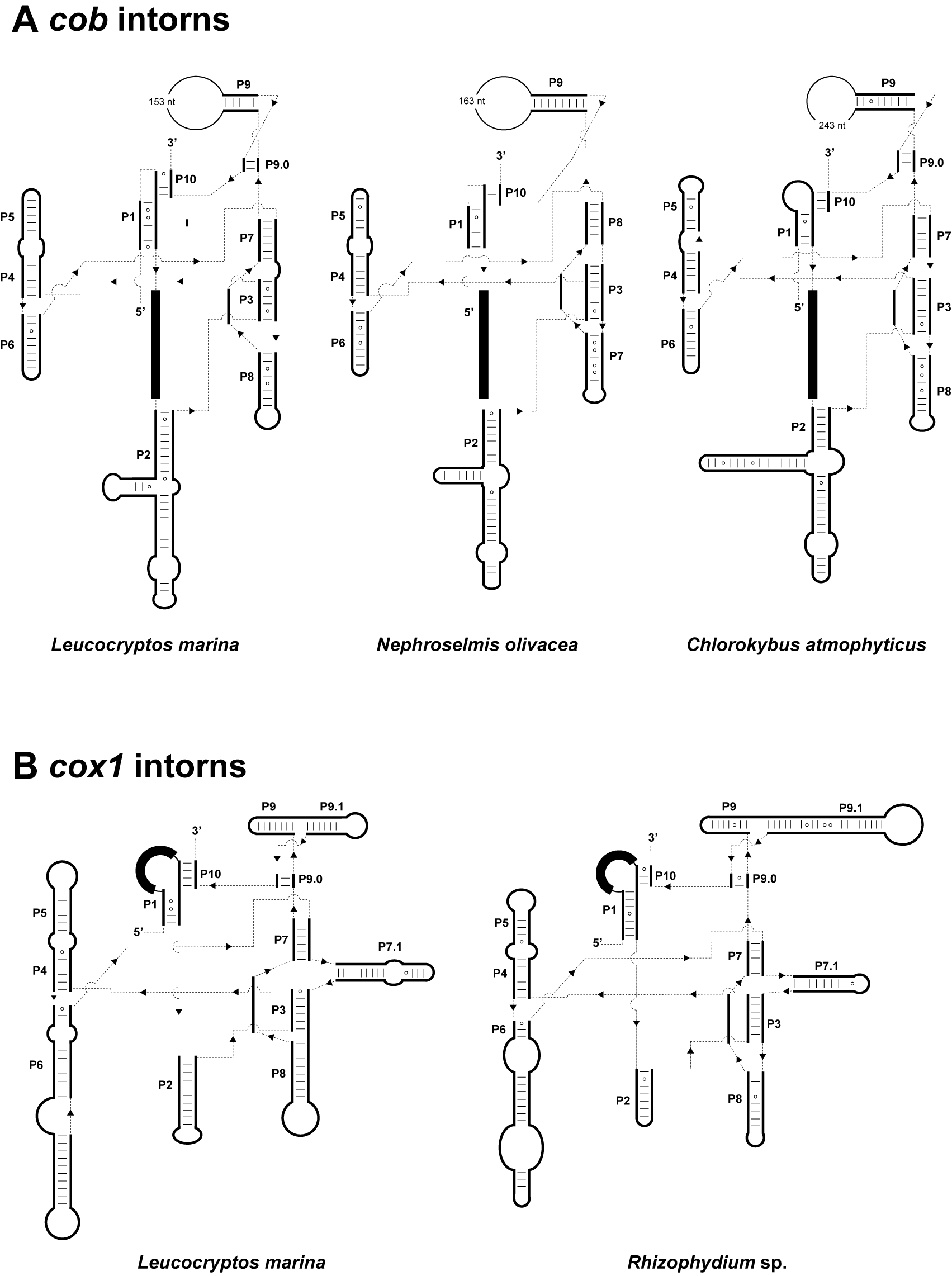

Supplement: Figure S1 — Putative secondary structures of group I intron RNAs. A. Schematic structures of Leucocryptos, Chlorokybus, and Nephroselmis cob introns. LAGLIDADG_2-type homing endonucleases are encoded in the region between P1 and P2 in the three introns (shown as closed boxes). B. Schematic structures of Leucocryptos and Rhizophydium cox1 introns. Both introns harbor LAGLIDADG_1-type homing endonucleases in the region between P1 and P10 (shown as closed boxes). (TIF) [file pone.0037307.s001.tif]

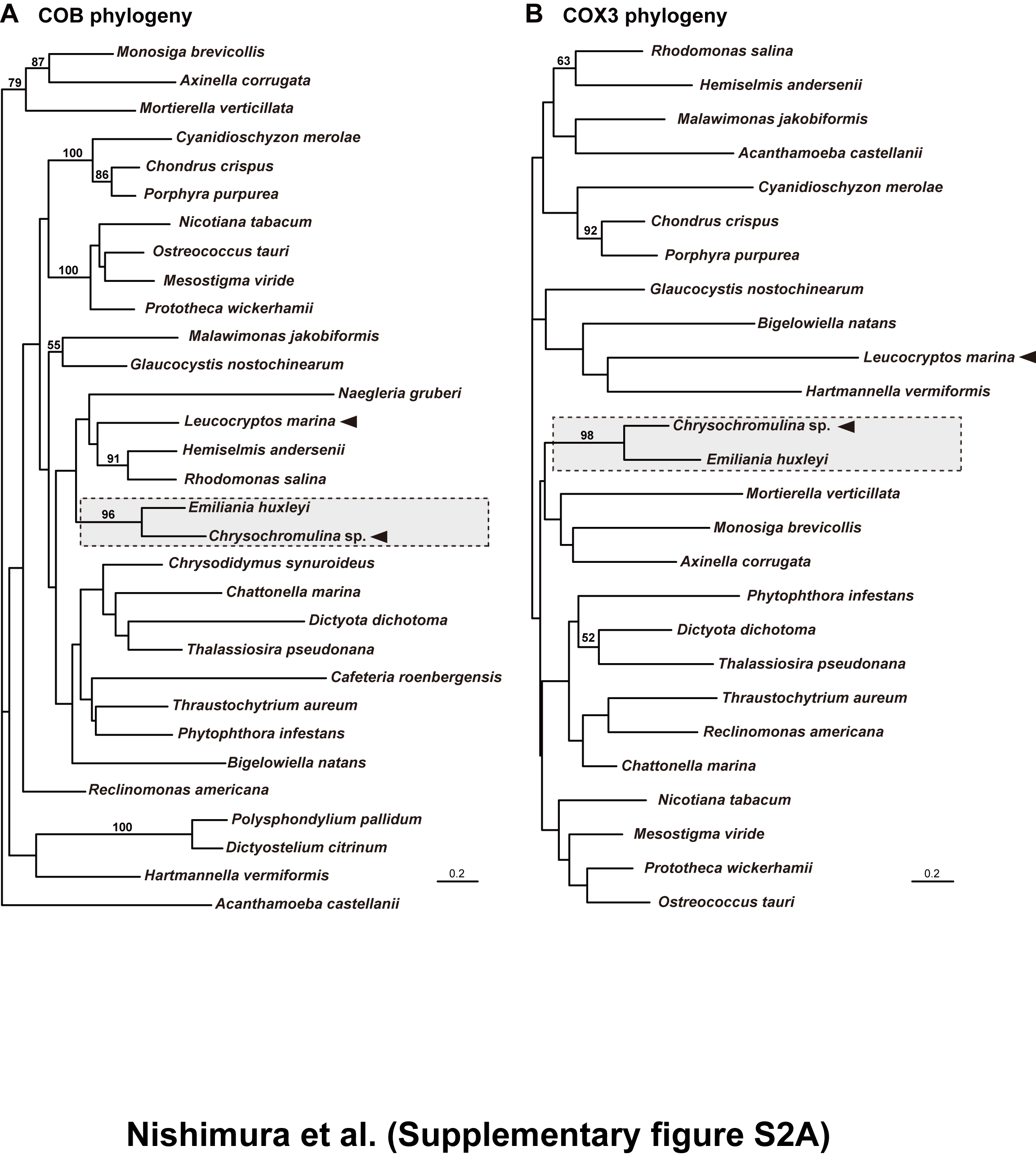

Supplement: Figure S2 — Maximum-likelihood (ML) analyses of the COB and COX3 amino acid (aa) alignments. A. The ML phylogeny inferred from the COB alignment comprises 31 taxa with 368 unambiguously aligned aa positions B. The ML phylogeny inferred from the COX3 alignment comprising 26 taxa with 218 unambiguously aligned aa positions. Leucocryptos marina and Chrysochromulina sp. are highlighted by arrowheads. The haptophyte clade is shaded. Only ML bootstrap values equal to or greater than 50% are shown. Methods: The two aa alignments were separately analyzed with the ML method with the LG+Γ+F model by using RAxML ver. 7.2.1. The details of the ML and ML bootstrap analyses were same as described in Materials and Methods/Phylogenetic analyses of intronic HEs. (TIF) [file pone.0037307.s002.tif]

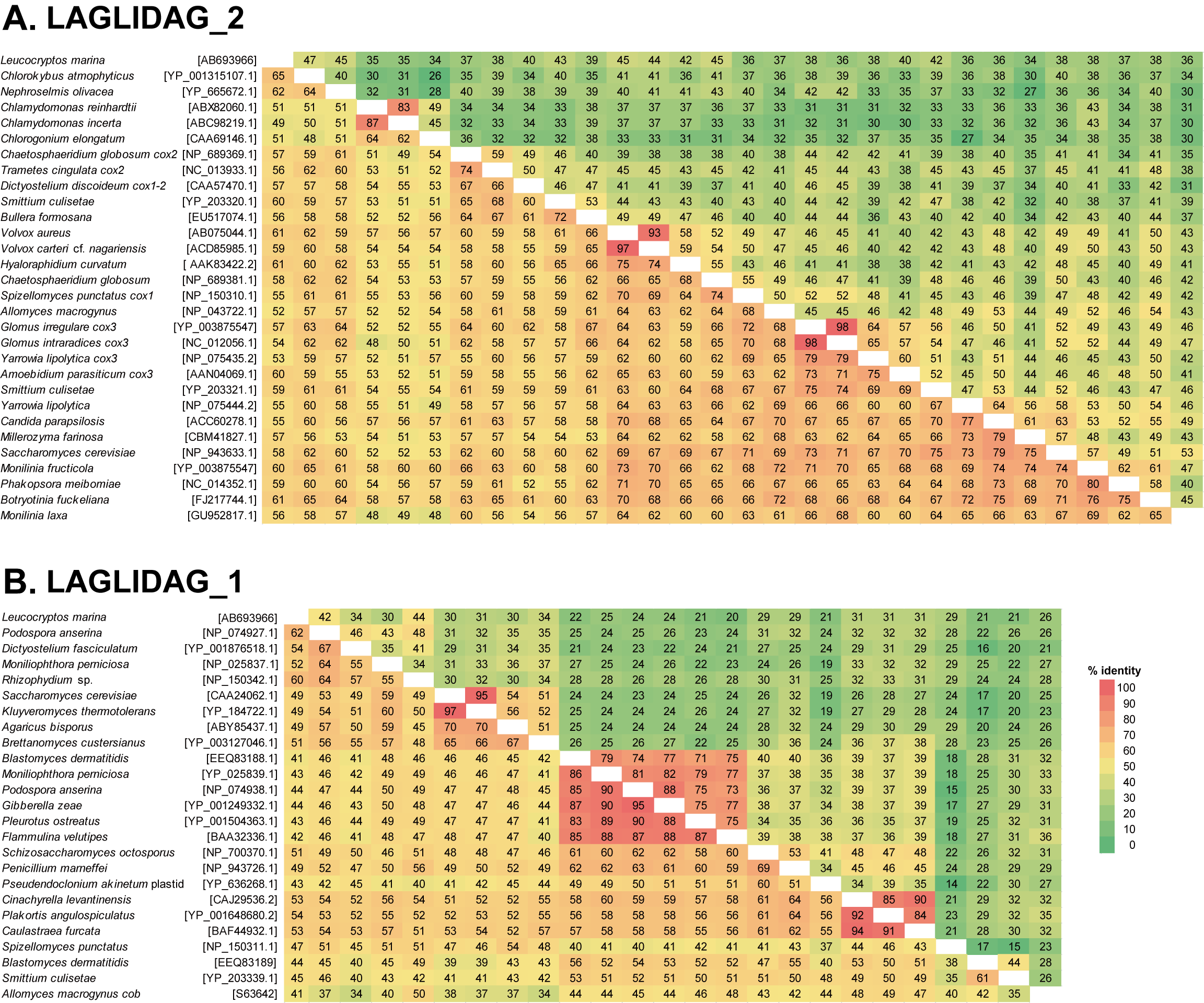

Supplement: Figure S3 — Amino acid (aa) sequence homology. A. Pairwise aa identity matrix of the 30 endonuclease (HE) sequences in the LAGLIDADG_2 alignment. We also recoded 20 aa characters in the HE sequences to six Dayhoff classes, and then made the identity matrix presented below diagonal. B. Pairwise aa identity matrix of the 25 HE sequences in the LAGLIDADG_1 alignment. We also provide the pairwise ‘Dayhoff-class’ identity matrix below diagonal. For each sequence, the GenBank accession no. is shown in brackets. (TIF) [file pone.0037307.s003.tif]
